# Correction: Duan et al. DCA-YOLOv8: A Novel Framework Combined with AICI Loss Function for Coronary Artery Stenosis Detection. *Sensors* 2024, *24*, 8134

**DOI:** 10.3390/s25175296

**Published:** 2025-08-26

**Authors:** Hualin Duan, Sanli Yi, Yanyou Ren

**Affiliations:** 1 School of Information Engineering and Automation, Kunming University of Science and Technology, Kunming 650500, China; 20222104102@stu.kust.edu.cn (H.D.); 20232204107@stu.kust.edu.cn (Y.R.); 2 Key Laboratory of Computer Technology Application of Yunnan Province, Kunming 650500, China

## Text Correction

There was an error in the original publication [1]. We would like to revise the content of the Institutional Review Board Statement. The manuscript does not involve data collection from human subjects. All experimental data are derived from publicly available datasets. The Institutional Review Board Statement is the work completed by the authors who provided the dataset; we did not undertake this task ourselves. The editor suggested that using “Not applicable” in the Institutional Review Board Statement is more accurate, and we fully agree with this recommendation.

A correction has been made to Institutional Review Board Statement:

Not applicable.

Meanwhile, the editor also suggested that we revise the content of the Informed Consent Statement, and we fully agree with this suggestion. Since the manuscript does not involve data collection from human subjects and all experimental data are derived from publicly available datasets, we did not undertake any related work in this regard.

A correction has been made to Informed Consent Statement:

Not applicable.

After careful review, we regret to find an error in the website link of the dataset provided in the manuscript. We would like to revise the content of the Data Availability Statement. Specifically, for Dataset II in the article, the link that should have directed to “https://data.mendeley.com/datasets/ydrm75xywg/1” (accessed on 12 November 2023) was mistakenly written as “https://data.mendeley.com/datasets/p9bpx9ctcv/2” (accessed on 12 November 2023). This error may cause confusion for readers when understanding the content of the article and, more importantly, interfere with the reproducibility of the experimental results, thereby affecting the verifiability of the research. This mistake occurred due to our failure to conduct thorough and meticulous verification during the final manuscript preparation and multiple proofreading stages, which constitutes a work oversight. We deeply apologize for the inconvenience this has caused to the original authors of the datasets used in this article.

A correction has been made to Data Availability Statement:

The data underlying the results presented in this study are available in Dataset I [37] (https://github.com/KarolAntczak/DeepStenosisDetection/tree/master/datasets). Accessed on 1 November 2023. The data underlying the results presented in this study are available in Dataset II [10] (https://data.mendeley.com/datasets/ydrm75xywg/1). Accessed on 12 November 2023. The code is available at https://github.com/duanhualin/DCA-YOLOv8.git (accessed on 1 November 2023).

The authors state that the scientific conclusions are unaffected. This correction was approved by the Academic Editor. The original publication has also been updated.
